# Effect of Acupotomy on FAK-PI3K Signaling Pathways in KOA Rabbit Articular Cartilages

**DOI:** 10.1155/2017/4535326

**Published:** 2017-10-12

**Authors:** Shi-Ning Ma, Zhan-guo Xie, Yan Guo, Jia-Ni Yu, Juan Lu, Wei Zhang, Li-Juan Wang, Jing Xu, Rui-Li Zhao, Shuai Zhou, Chang-Qing Guo

**Affiliations:** ^1^School of Acupuncture-Moxibustion and Tuina, Beijing University of Chinese Medicine, Beijing 100029, China; ^2^The First People's Hospital of Dongcheng District, Beijing 100075, China; ^3^First Hospital Affiliated to Tianjin College of Traditional Chinese Medicine, Tianjin 300193, China; ^4^The Second Affiliated Hospital of Guangdong University of Traditional Chinese Medicine, Guangdong 510120, China

## Abstract

**Objective:**

By observing the needle-knife of KOA rabbit morphology, knee joint cartilage p-FAK, p-PI3K, Aggrecan gene, and protein expression, to study the effect of needle-knife to promote cartilage cell synthesis metabolism mechanism.

**Method:**

49 male New Zealand rabbits, randomly divided into normal group (Z), model group (M), model-inhibitors (MP), needle-knife group (D), needle-knife inhibitors group (DP), electroacupuncture group (E), and electroacupuncture inhibitors (EP). RT-PCR and Western Blot were used to test each animal cartilage p-FAK, p-PI3K, and Aggrecan gene and protein expression level.

**Results:**

Compared with N group, p-FAK and p-PI3K protein and mRNA expression of M group, D group, and E group increased (*P* < 0.05), while the protein and mRNA expression of Aggrecan reduced (*P* < 0.05). Compared with M group, p-FAK, p-PI3K, Aggrecan protein, and mRNA of E and D group increased (*P* < 0.05). Compared with E group, p-FAK, p-PI3K, Aggrecan protein, and mRNA expression of D group increased (*P* < 0.05); after adding inhibitors, p-FAK, p-PI3K, Aggrecan protein, and mRNA expression reduced (*P* < 0.05).

**Conclusion:**

Needle-knife therapy can promote the repairment of cartilage cells by activating FAK-PI3K signaling pathways, promoting the synthesis of cartilage cell metabolism.

## 1. Introduction

Knee osteoarthritis (Knee Osteoarthritis KOA) takes pain, stiffness and functional disorder as the main clinical manifestations, and cartilage degeneration as typical pathological change [1]. Often called wear-and-tear arthritis, KOA is a knee joint degenerative disease that affects soft tissues around the knee including the muscle, tendon and joint capsule [2]. According to epidemiological survey, for middle-aged and elderly people in china, the incidence rate of KOA is about 20% [3, 4]. KOA affects nearly 8 million people in the United Kingdom and about 27 million people in the United States [5].

In serious condition, it could lead to joint deformities and even the loss of joint function, thereby affecting patients' life quality and mobility and it is linked with an excess mortality [6–13]. While genetics, aging, obesity, injury, and biomechanical stress are considered as the main risk factors involved in the pathogenesis of OA, obesity is the primary preventable risk factor for OA [14–18]. Obesity increases the risk of developing OA in both weight-bearing joints (especially the knee) and non-weight-bearing joints (the hand) [15], indicating that obesity-related mechanical and nonmechanical factors increase the risk of OA. In addition, the KOA is more common in women than in men, implying that differences in sex hormones modulate the disease, and the effect of oestrogen replacement therapy may protect against incident of OA in postmenopausal women [19–21].

As a disease of the entire knee joint, KOA can originate not only from degenerative changes of cartilages and bones (such as cartilage wearing, subchondral bone lesions, and osteophytes) but also from tears and subluxation of menisci, sprain of ligaments, synovitis, etc. [17, 22–24]. It is becoming clear that articular tissues other than cartilage play an important role in the process of OA.

Because the causes behind OA development and progression continue to remain largely undefined, understanding the molecular pathogenesis of the disease remains a priority [25]. Experimental researches proved that inflammatory cytokines, free radical, chondrocyte apoptosis and metabolism, protease, and inhibitor were all involved in the pathogeny of KOA [26].

Besides well-known molecules of mediating articular cartilage destruction, such as tumor necrosis factor-a (TNF-a), interleukin-1 (IL-1), and interleukin-6 (IL-6), the human cartilage glycoprotein chitinase 3-like-1 (CHI3L1), lubricin, and *β*-defensins-4 are also introduced into the pathophysiological process (repairing) of OA recently [27–29]. Recent studies have shown that the levels of CHI3L1 may be particularly involved in the pathogenesis of OA. Produced by articular chondrocytes, synoviocytes, and macrophages, CHI3L1 is associated with the cartilage damage and mediators of inflammation [30–33]. Lubricin has been found in several tissues including the synovial membranes and SF, the superficial zone of articular cartilage, tendon and ligament, disc, and meniscus. Maintaining joint integrity, lubricin prevents cartilage wear and synovial cell adhesion and proliferation and reduces the amount of friction of the articular cartilage surface [34]. Recently studies demonstrated that the lack of lubricin secretion may be involved in the pathology of OA [35]. It is also reported that CHI3L1 and lubricin are two glycoproteins that are functionally associated with the development of OA and in particular with grade 2/3 of OA [25].

However, as the core pathological change of KOA, cartilage degradation has always been the focus of KOA research [36]. Many clinical studies have demonstrated that needle-knife is simple, inexpensive, effective, and safe in treatment of KOA [37–40]. In our previous animal experiments, we found that needle-knife intervention can improve gene and protein expression of cartilage integrin beta 1 (integrin beta 1) and repair cartilage [41]. Through analyzing needle-knife's effect on morphology and gene and protein expression of p-FAK, p-PI3K, and Aggrecan in knee articular cartilage of KOA rabbit, this study explores the mediating pathway through which needle-knife repairs the cartilage and to further improve the theoretical and experimental basis of this therapy for KOA.

## 2. Material

### 2.1. Animals Preparation

49 New Zealand white rabbits aged 6 months, weight 2.5 ± 0.54 kg, bought from Beijing Jin Muyang Laboratory Animal Technology Co., Ltd., animal batch number: SCXK Beijing 2015-0005, raised in Beijing military region general hospital orthopedic experiment center, the experimental animals housed separately in single cage, which are fed and watered freely and which are provided with natural light. The temperature in the room remained at 22 ± 2°C, and the humidity is 40~60%, disinfecting the laboratory by ultraviolet on a regular basis.

### 2.2. The Experimental Materials

The experimental materials are GuBao resin bandage (specification: 15 cm × 180 cm, ZhuHai LiZhu Medical Biological Material Co., Ltd.); macromolecule resin bandage (specification: 7.5 cm × 360 cm, Suzhou Knight Medical Technology Co., Ltd.); HZ series disposable needle-knife (specification: 0.4 × 40 mm, Beijing Outstanding Huayou Medical Instrument Co., Ltd.); Zhongyan Taihe disposable acupuncture needle (specification: 0.25 × 25 mm, Beijing Research Taihe Pharmaceutical Co., Ltd.).

### 2.3. The Instrument

The instruments are as follows: HANS acupoint nerve stimulator, LH202H type, Beijing Huawei Industry Development company; the electrophoresis of protein and the membrane system, the USA Bio-Rad company; full-wavelength multifunctional enzyme mark, Tecan Austria Co., Ltd., models: Infinite the M1000; gel imaging system, the Alpha Innotech® company, models: Fluor Chem FC2; PCR instrument type 5020, Finland thermoelectric; PCR C1000, the United States bole; ultraviolet spectrophotometer, UV-2600, the German Eppendorf company; scanning electron microscope (sem): Quanta 250, Czech republic, FEI; manual magnetron sputtering coating machine: SC7620, Britain, the Quorum; critical point drying apparatus: K850, Britain, the Quorum.

### 2.4. The Reagent

The reagents are as follows: PF-562271, the item number: S2890, Selleck company; P-FAK rat rabbit monoclonal antibody, the item number: FM1211, ECM Bioscience companies in the United States; P-PI3K sheep rabbit polyclonal antibody, the item number: sc-12929, Santa Cruz companies in the United States; Aggrecan rat rabbit monoclonal antibody, the item number: NB600-504, Novus companies in the United States; TRIzol reagent, the item number: 15596-026, Invitrogen™ ∣ Thermo Fisher Scientific; reverse transcription cDNA kits, the item number: k1622, Invitrogen ∣ Thermo Fisher Scientific; Power SYBR Green, the item number: 4367659, ABI; PCR primers, Invitrogen ∣ Thermo Fisher Scientific synthesis; glutaraldehyde, SPI companies in the United States; 4% paraformaldehyde: Beijing Chemicals company; 15% EDTA, Beijing Chemical Reagents company; osmic acid, SPI companies in the United States; acetone, Beijing East Hair Lue Petroleum Chemical Industry Co., Ltd.; iso-amyl acetate, Shanghai Vibration Spectrum Biological Technology Co., Ltd; ethanol, Shengyang Moist (Beijing) Technology Co., Ltd.; liquid carbon dioxide, Beijing Yanglilai Chemical Gas Co., Ltd.; 0.1 mol/L PH 7.2 phosphate buffer, Beijing KYKY instrument Technology Development Co., Ltd.

## 3. Methods

### 3.1. Animal Grouping

49 New Zealand rabbits were randomly divided into blank control group, model group, model-inhibitor group, needle-knife group, needle-knife inhibitor group, electroacupuncture group, and electroacupuncture inhibitor group by random number table method, 7 rabbits in each group. Six rabbits were used for the final index detection, and one was used to determine success of model preparation with morphological detection. In the course of the experiment, the animals are disposed strictly according to the Ethical Committees on Animal Committees on Animal Research (Animal Experimental Ethical Inspection Form Number Kj-dw-18-20150604-01), with approval in National Natural Science Foundation (number 8157150725).

### 3.2. Model Preparation

Animals except those of the blank control group were prepared as KOA models by improved Videman's method, which fixed the left hind limb in extension position: rabbits intended for model preparation were fasted 10–16 hours and anesthetized with 3% pentobarbital sodium (30 mg/kg) through ear edge vein injection. After anesthesia, the rabbits were fixed on the workbench in supine position, with left hind limb in full extension. The left hind limb was fixed from the groin to toe by softened resin bandage (softened by hot water at 65–85 degrees centigrade), with absorbent cotton on the inner side of the bandage. The ankle joint was dorsiflexed to 60 degrees, and knee joint was fixed in straight position. Resin bandage was fixed by the medical gauze and then wrapped with two layers of macromolecule resin bandage. Silk stocking was pulled on the bandage after the bandage was set to protect it from animal bite. The stocking was fixed with medical tape and partly cut to expose the toes for observing blood supply and swelling. Motion of animals' left hind limb was constrained by the bandages and stocking for 5 weeks for model preparation. After that, one rabbit in each group was sacrificed and the articular cartilage was taken for pathological examination. The bandage and stocking of all animals were removed after we proved the success of model preparation.

### 3.3. Interventions

① Blank control group: there was no intervention. ② Model group: there was no intervention after model preparation. ③ Needle-knife group: needle-knife intervention was applied one week after model preparation. After routine skin preparation and disinfection, needle-knife was pierced and then withdrawn, with the point pressed to stop bleeding. The intervention was given 2 times per week and lasted for 4 weeks. The specific operation on each piercing point is as follows: (1) insertion of needle-knife in tendons of medial vastus muscle and musculus vastus lateralis: to release tendon extension of medial vastus muscle and musculus vastus lateralis. Insert the needle-knife perpendicular to the muscle belly with the edge of needle-knife parallel to the tendon. After releasing to the direction of tendons and bone connection, the needle-knife was withdrawn with the point pressed for a while. (2) Insertion of needle-knife in tendon of rectus femoris: to release upper edge of patella, where there is the junction of tendon of rectus femoris and the patella. Insert the needle-knife perpendicular to the muscle belly with the edge of needle-knife parallel to the tendon. After releasing to the direction of tendons and bone junction, needle-knife was withdrawn with the point pressed for a while. (3) Insertion of needle-knife in tendon of biceps femoris: to release the tendon of biceps femoris. Insert the needle-knife perpendicular to the muscle belly with the edge of needle-knife parallel to the tendon. After releasing to the direction of tendons and bone junction, the needle-knife was withdrawn with the point pressed for a while. (4) Muscular fiber trabs and nodules: to search and locate fiber trabs and nodules around knee joint by palpation. And then insert the needle-knife perpendicularly with the edge of the needle-knife parallel to the fiber trabs or nodules. After releasing along the fiber trabs, the needle-knife was withdrawn with the point pressed for a while. ④ E group: one week after the removal of fixation, Liang qiu (ST 34), Xue hai (SP 10), Nei xi yan (EX-LE4), and Wai xi yan (EX-LE5) were selected for needling. After insertion of needles, rarefaction-dense wave was applied with a frequency of 2/100 Hz and current of 3 mA by Han's acupoint nerve stimulator. Two circuits were formed by connecting Liang qiu (ST 34) with Xue hai (SP 10) and Nei xi yan (EX-LE4) with Wai xi yan (EX-LE5). The treatment lasted for 20 mins each time, 3 times per week and 4 weeks in all. ⑤ In these three groups, model-inhibitor group, needle-knife inhibitor group, and electroacupuncture inhibitor group, PFK-specific inhibitor PF-562271 (200 *μ*M, 0.5 ml) was injected intra-articularly 2 hours before each treatment, and other operations are the same with the corresponding model group, needle-knife group, and electroacupuncture group.

### 3.4. Sample Obtaining

All the animals were then sacrificed by overdose of anesthesia. Knee joint cavity of right hind limb was opened. And ligament and other soft tissues around the knee joint, including anterior and anterior cruciate ligament, were cut off. Meniscus was cut off to expose the cartilage of distal femur and proximal tibia. A 1*∗*1 cm cartilage specimen of the medial condyle of the femur was obtained with rongeur. Samples were flushed with saline to wash away blood, mucus, and tissue debris and then fixed in 4% poly formaldehyde solution for 72 h for subsequent index detection. Adequate cartilage tissues were cut by surgical blade, collected in freezing tube, and stored in −80°C refrigerator for Western Blot and PCR real-time detection after being quick-frozen in liquid nitrogen.

### 3.5. Index Detection

#### 3.5.1. Scanning Electron Microscope Observation

Sample preparation: fixed specimens were rinsed by 0.1 mol natrium cacodylicum buffer solution and fixed in 1% osmium tetroxide for two hours. Then they were rinsed by 0.1 mol natrium cacodylicum buffer solution and dehydrated by ethanol, then kept in isoamyl acetate for one night. After dying by carbon dioxide critical point dying method, the specimens adhered to objective table, were sprayed with AU coating by vacuum sputter, and were observed and photographed with scanning electron microscope.

#### 3.5.2. Detection of p-FAK, p-PI3K, and Aggrecan Gene Expression with RT-PCR

Total RNA was extracted from the crushed cartilage tissue with TRIzol reagent. RNA concentration was calculated according to the OD value of the extracts. Sample solution containing 5 *μ*l RNA stock solution was mixed with 1 *μ*l Oligo dT, 4 *μ*l 5x reaction buffer, 1 *μ*l RiboLock RNase inhibitor, 2 *μ*l 10 mM dNTP Mix, and 1 *μ*l RevertAid M-Mul V RT to perform reverse transcription and acquire cDNA. Then 2 *μ*l cDNA was mixed with 0.2 *μ*l upstream primer and 0.2 *μ*l downstream primer, 7.2 *μ*l RNAse-free water, and 10 *μ*l Power SYBR Green for amplification. The obtained data was analyzed by relative quantitative (2^−ΔΔCT^) method. ΔCT = Ct value of target gene minus Ct value of control gene (GAPDH) of the same sample. −ΔΔCT = ΔCT minus the mean ΔCT value of the blank group. The expression of target gene of each sample was calculated as 2^−ΔΔCT^.

#### 3.5.3. Western Blot Method for Detection of Protein Expression of p-FAK, p-PI3K, and Aggrecan

The total protein of cartilage tissue was extracted by cell lysis method. Protein concentration was tested by BCA method. After SDS-PAGE electrophoresis and nitrocellulose filter transfer, antigen-antibody reaction by primary antibody and secondary antibody, and color rendering by ECL, Alpha Innotech Fluor Chem FC2gel imaging system was used to acquire image and ImageJ software was applied for analyzing the optical density value of each target protein. The ratio of optical density value of each target protein to optical density of beta-actin was used for statistical analysis.

### 3.6. Statistical Analysis

The data were analyzed by SPSS 21.0 statistical software. For the data with normal distribution and homogeneity of variance, single-factor analysis of variance was used to compare the data among groups. LSD-*t*-test was used to compare the two groups. For the data accorded with normal distribution, rather than homogeneity of variance, Tamhane's T2 method was used to compare the data among groups. The sample data for each group is expressed by the mean ± standard deviation $\left(\overset{-}{x}\pm s\right)$, with *P* < 0.05 for a significant difference and *P* < 0.01 for a very significant difference.

## 4. Results

### 4.1. Observation of Cartilage by Scanning Electron Microscope

**Figure 1:**
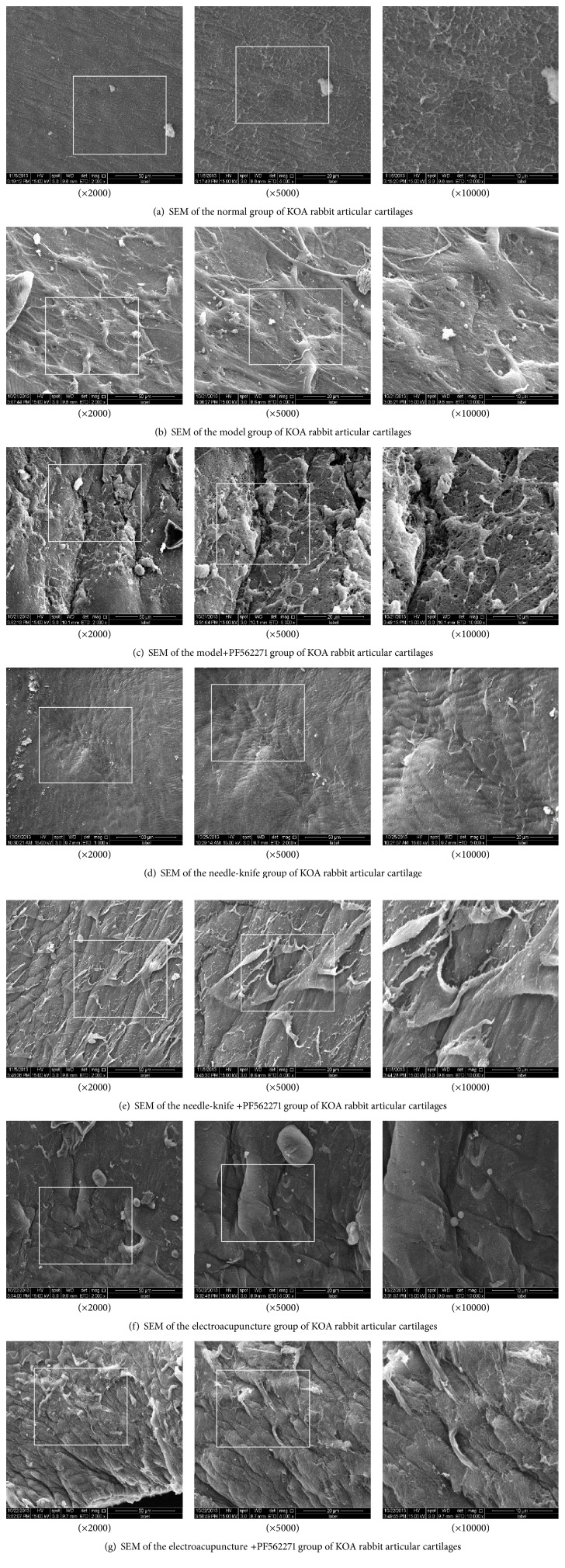


Scanning electron microscope showed that the surface of knee articular cartilage of normal group rabbits was smooth. There are small uplifts on surface of the cartilage with furrows paralleled to it. The surface of cartilage was evenly covered with amorphous substance, indicating complete structure of the superficial layer (Figure 1(a) (×2000), Figure 1(a) (×5000), and Figure 1(a) (×10000)).

Scanning electron microscope showed that the surface of knee articular cartilage of model group rabbits was not smooth. The structure of small uplifts on surface of the cartilage with furrows disappears, with collagen fibers in sublayer enlarged, in the branches of the sample distribution, irregular arrangement (Figure 1(b) (×2000), Figure 1(b) (×5000), and Figure 1(b) (×10000)).

Surface of knee articular cartilage of model inhibitor group rabbits was seriously damaged. There are maybe cracks on the surface of the sample. Serious desquamation of gelatinous substance can be seen on the surface of the sample material. Collagen fibers reduced also (Figure 1(c) (×2000), Figure 1(c) (×5000), and Figure 1(c) (×10000)).

Surface of knee articular cartilage of needle-knife rabbits was smooth. The furrows got deeper and were regularly arranged. However, no obviously parallel arrangement between furrows was observed. Cartilage surface was evenly covered by amorphous substance. But small cracks of the amorphous substance can be observed all over the cartilage surface. Exposed collagenous fiber can occasionally be observed (Figure 1(d) (×2000), Figure 1(d) (×5000), and Figure 1(d) (×10000)).

Scanning electron microscope showed that the surface of knee articular cartilage of needle-knife-inhibitor group rabbits was not smooth. The furrows got deeper and were regularly arranged. However, no obviously parallel arrangement between furrows was observed. Collagen fibers were turned up with enlargement of the sample distribution (Figure 1(e) (×2000), Figure 1(e) (×5000), and Figure 1(e) (×10000)).

Knee articular cartilage surface of electroacupuncture group rabbits was of rough surface. And the furrows were getting deeper and increased in number, no obviously parallel arrangement. Collagen fibers were turned up (Figure 1(f) (×2000), Figure 1(f) (×5000), and Figure 1(f) (×10000)).

Scanning electron microscope showed that the surface of knee articular cartilage of EA-inhibitor group rabbits was of rough surface. The small uplifts were deepened and widened, with a rock sample appearance. There are visible fibrinoid structure and irregular collagen fibers (Figure 1(g) (×2000), Figure 1(g) (×5000), and Figure 1(g) (×10000)).

### 4.2. Gene and Protein Expression of p-FAK, p-PI3K, and Aggrecan in Cartilage

After model establishment, gene expression of p-FAK and p-PI3K increased in model group, needle-knife group, and E group (*P*^*∗*^ < 0.05 or *P*^*∗∗*^ < 0.01); compared with model group, gene expression of p-FAK and p-PI3K increased after treatment in needle-knife group and E group (*P*^#^ < 0.05 or *P*^##^ < 0.01); compared with E group, gene expression of p-PI3K increased in needle-knife group (*P*^▲^ < 0.05); when inhibitor was applied, gene expression of p-FAK and p-PI3K decreased (*P*^Δ^ < 0.05 or *P*^ΔΔ^ < 0.01). Meanwhile, compared with control group, gene expression of Aggrecan decreased in model group, needle-knife group, and E group (*P*^*∗*^ < 0.05 or *P*^*∗∗*^ < 0.01); compared with model group, gene expression of Aggrecan increased after treatment in needle-knife group and E group (*P*^#^ < 0.05 or *P*^##^ < 0.01); compared with E group, gene expression of Aggrecan increased in needle-knife group (*P*^▲▲^ < 0.01); when inhibitor was applied, gene expression of Aggrecan decreased (*P*^Δ^ < 0.05 or *P*^ΔΔ^ < 0.01).

### 4.3. Protein Expression of p-FAK, p-PI3K, and Aggrecan in Cartilage

**Figure 2:**
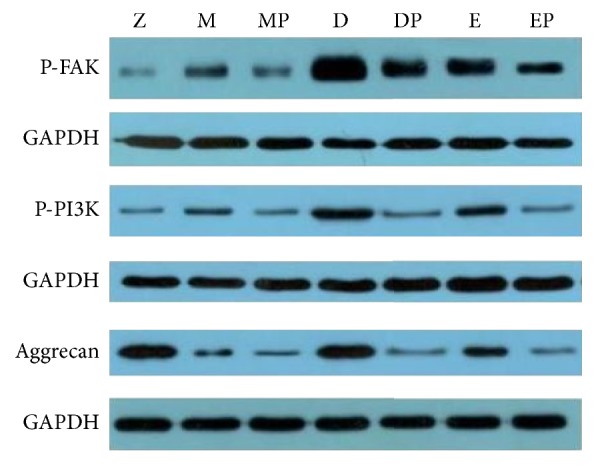
Protein expression stripe of FAK, PI3K, and Aggrecan.

Compared with normal group, protein expression of p-FAK, p-PI3K, and Aggrecan increased in different degrees after treatment in model group and in needle-knife group and E group (*P*^*∗*^ < 0.05, *P*^*∗∗*^ < 0.01); compared with model group, protein expression of p-FAK and p-PI3K increased in needle-knife group and E group (*P*^#^ < 0.05, *P*^##^ < 0.01); compared with E group, protein expression of p-FAK and p-PI3K increased in needle-knife group (*P*^▲▲^ < 0.01); when inhibitor was applied, protein expression of p-FAK and p-PI3K decreased and compared with their respective noninhibitors group was statistically significant (*P*^Δ^ < 0.05). Meanwhile, compared with control group, protein expression of Aggrecan decreased in model group, needle-knife group, and E group (*P*^*∗∗*^ < 0.01); compared with model group, protein expression of Aggrecan increased after treatment in needle-knife group and E group (*P*^#^ < 0.05, *P*^##^ < 0.01); compared with E group, protein expression of Aggrecan increased in needle-knife group (*P*^▲▲^ < 0.01); when inhibitor was applied, protein expression of Aggrecan decreased and compared with their respective noninhibitors group was statistically significant (*P*^Δ^ < 0.05, *P*^ΔΔ^ < 0.01) (Figure 2, Tables 1 and 2).

## 5. Discussion

KOA takes cartilage damage as the core of its pathological change [42, 43]. Cartilage is a viscoelastic structure composed of cartilage matrix and cartilage cells [44]. It bears mechanical load and absorbs and cushions stress. The functions of cartilage mainly depend on the cartilage matrix. Cartilage cells synthesize and secrete the essential components which maintain the integrity of cartilage matrix, such as proteoglycan (Aggrecan). Under physiological conditions, there is a dynamic balance between the synthesis and decomposition of chondrocytes, which is the basic guarantee of the normal structure of cartilage matrix [45]. When suffering from KOA, decomposition of chondrocytes dominates metabolism of chondrocytes and breaks the homeostasis. As a result, degradation of cartilage finally occurred. The stress is one of the important factors for maintenance of the health of cartilage due to its special physiological structure [46].

In recent years, studies have found that moderate dynamic mechanical load is a potential method for the prevention and treatment of KOA, although both insufficient and excessive stress of the knee joint will induce cartilage degradation. Its therapeutic effects of inhibiting inflammatory reaction and maintaining balance of chondrocyte metabolism may be achieved through some mechanical transduction pathways that are not yet clear [47]. Studies have shown that moderate dynamic mechanical loads could obviously reduce inflammation and protect ECM. They inhibit expression of proinflammatory cytokines such as IL-1*β*, IL-6, and TNF-*α* and inflammatory mediators such as COX-2, PGE2, and NO and promote expression of anti-inflammatory cytokines such as IL-4 and IL-10 to exert anti-inflammatory effects. Meanwhile, they protect ECM by suppressing the expression of MMPs, disintegrin, and ADAMTS [48]. The special mechanical receptor on the surface of chondrocytes and the mechanical transduction pathway induced by it played a key role in the above process. O'Conor et al. proved that direct moderate stress stimulation to the chondrocytes can promote synthesis of chondrocytes and alleviate degradation of cartilage matrix [49]. Therefore, benign stress can not only maintain the metabolic balance of healthy cartilage but also promote the recovery of injured cartilage. In previous studies, we confirmed that needle-knife treatment can promote the synthesis of chondrocytes to some extent and repair the damage of cartilage. After needle-knife treatment, the expression of Aggrecan protein of extracellular matrix of KOA rabbits' knee cartilage increased, together with the upregulation of protein expression of integrin beta one, which is the most important mechanical receptor on cartilage [50]. Therefore, the mechanism of cartilage repair by needle-knife is closely related to the improvement of stress balance of knee joint. However, the underlying signal pathway is unknown.

Integrin is one of the mechanical receptors on cell surface. The mechanical receptors on the surface of chondrocyte are mainly integrin, a kind of transmembrane protein that consists of alpha and beta subunits [51]. With its extracellular domain connecting to matrix ligand and its intracellular domain interacting with kinases of intracellular pathways to induce cascade reaction, the integrin transduces extracellular signal into the cell to complete the process of transduction from mechanics to chemical function, thereby regulating the proliferation, differentiation, survival, and migration of chondrocytes and morphogenesis and remodeling of tissues. In the transduction process, focal adhesion kinase (FAK) is a key material of signal pathway mediated by integrin. It works like an integrated device that receives and integrates the signal carried by integrin and then amplifies it within the cell. As a result, it activates many downstream signal pathways, like phosphatidylcholine-3 kinase (PI3K), MAPK pathway, and so on [52].

FAK is a cytoplasmic nonreceptor protein tyrosine kinase [53]. It is a member of focal adhesion complexes family and consists of 1028 amino acids [54]. The structure of FAK can be divided into three functional areas including N-end function area, C-end function area, and kinase region [50]. N-end function area mediates the interaction between FAK and integrin protein and the kinase region is the catalytic site of FAK activation. C-end function area can make FAK adhere to focal adhesion, forming focal adhesion complexes to make FAK play its role [55]. When the integrin was stimulated by extracellular mechanical stimulation, its configuration changed followed by the recruitment of FAK to intracellular region of the integrin and the initiation of self-phosphorylation of Tyr397. Tyr397 self-phosphorylation induces its combination with SH2 structural domain of intracellular tyrosine kinase (Src) and the formation of Src-FAK complex, leading to the activation of Src. The activated Src can catalyze the phosphorylation of Tyr576/577 of FAK, thereby completely activating FAK. The fully activated FAK will further activate PI3K by binding to its P58 subunit with site Tyr397 [56, 57]. The activated PI3K can produce the second messengers, phosphatidylinositol 3,4,5-triphosphate, to alleviate the degradation of cartilage by synthetizing and secreting important components of cartilage, such as proteoglycan [58].

In our study, protein and gene expression of p-FAK, p-PI3K, and Aggrecan increased in different degrees after treatment in needle-knife group and E group but obviously decreased when specific inhibitor for FAK was applied. Furthermore, the effect of needle-knife on protein expression of p-FAK, p-PI3K, and Aggrecan is better than that of EA treatment. Meanwhile, the result of cartilage scanning electron microscope showed that cartilage condition of rabbits in both needle-knife and E group improved after treatment, compared with that of the model group, and needle-knife treatment has a better effect. This result is consistent with test results of protein and gene expression. The above results showed that both needle-knife and EA can activate FAK-PI3K pathway, promote the synthesis of chondrocytes, increase the expression of Aggrecan, and finally promote the recovery of cartilage. Needle-knife has a better effect than EA. We believe that this effect is a result of activation of FAK-PI3K mechanical pathway, which plays an important mediating role in the recovery of cartilage. Furthermore, the mechanism of cartilage recovery by needle-knife is related to the restoration of benign stress of knee joint.

In this study although the PROM and motor function were improved, we still cloud not explain how the compressive stress of cartilage surface changes. Hence it is advisable to do cartilage compressive stress and related mechanical experiments of knee joint cartilage in order to improve the theoretical research on KOA biomechanical mechanism with needle-knife.

Clinically, on the early stage of KOA, patients are usually treated by acupuncture and functional exercises by one of the first authors (Shi-Ning Ma). With the pathological development of KOA, the needle-knife is necessary for loosening tissue adhesion mechanically when organic injuries (such as tissue hyperplasia, hypertrophy, and fibrosis) take place. Generally, the KOA patients' subjective satisfaction and subjective functional improvement present a positive correlation in the needle-knife treatment. Sometimes, subjective and objective evaluations do not entirely chime with each other [59]. Usually, a few patients who could perform the daily living activity (such as walking, stair activity) originally before treatment are not very satisfied, though there are improvements on objective scores after treatment. However, there are still some patients that are extremely pleased with the functional recovery of daily living activity despite limited score improvement after treatment.

The aseptic operation is crucial in the clinical treatment, and both acupuncture and needle-knife are invasive operations. No matter how small the incision is, even the perpendicular needling of acupuncture operation, there is still a certain potential risk of infection. The risks of secondary infection after operation will increase significantly especially on the weak elderly, the immunocompromised subjects, the infectious subjects, and the patients with chronic diseases [60]. Because the acupotomy is the result of combination of Traditional Chinese Medicine (TCM) and Western Medicine, strict sterile manipulation is the basic requirement which could avoid the risk of infection to the utmost extent.

Clinical painless operation is another important issue that we should be pay attention to [60, 61]. With a similar puncturing method and surgical approach to traditional acupuncture, the pain stimulus produced by the special needling instrument of acupotomy is acceptable for most patients. Therefore, communicating well with patients in order to eliminate patients' fear to most is necessary before operation.

Actually, the needle-knife is not the real meaning of “knife.” The difference between needle and knife is not only in the difference of shape but also in the difference of function. The function of needle is puncturing while the function of knife is cutting. Both of the aims of needle and knife operation are amputation of tissues, though their quantities of amputation are in different degree [62]. Thus there are no essential differences, but there is an obvious quantitative difference between them. Both of acupuncture and needle-knife belong to per cutem inserting needle method. Avoiding the incision of skin method, the per cutem inserting needle method of acupotomy could minimize the operation injury in the anatomical structure. Because of the specific bit tool of needle-knife, the local lesion tissues could be needled, cut, stripped, and shoved. The clinical treatment effect of needle-knife could not be achieved by traditional acupuncture on KOA.

## Supplementary Material

Animal Experimental Ethical Inspection Form, Beijing University of Chinese Medicine.

## Figures and Tables

**Table 1:** Gene expression of FAK, PI3K, and Aggrecan of KOA rabbit articular cartilages ($\overset{-}{x}\pm s$).

Group	*N*	Gene expression of FAK	Gene expression of PI3K	Gene expression of Aggrecan
Normal	6	1.01 ± 0.19	1.02 ± 0.21	1.01 ± 0.17
Model	6	1.30 ± 0.10^*∗*^	1.22 ± 0.21^*∗*^	0.55 ± 0.07^*∗∗*^
Model-inhibitor	6	1.05 ± 0.08^Δ^	1.09 ± 0.08	0.38 ± 0.05^Δ^
Needle knife	6	1.57 ± 0.20^*∗∗*#^	1.46 ± 0.17^*∗∗*#▲^	0.76 ± 0.07^*∗∗*##▲▲^
Needle knife-inhibitor	6	1.25 ± 0.13^ΔΔ^	0.98 ± 0.11^ΔΔ^	0.51 ± 0.07^ΔΔ^
Electroacupuncture	6	1.47 ± 0.33^*∗∗*^	1.25 ± 0.24^*∗*^	0.61 ± 0.06^*∗∗*#^
Electroacupuncture inhibitor	6	1.06 ± 0.10^ΔΔ^	0.84 ± 0.12^ΔΔ^	0.43 ± 0.08^ΔΔ^

Versus normal group: *P*^*∗*^ < 0.05 and *P*^*∗∗*^ < 0.01; versus model group: *P*^#^ < 0.05 and *P*^##^ < 0.01; versus electroacupuncture group: *P*^▲^ < 0.05 and *P*^▲▲^ < 0.01; versus noninhibitor group: *P*^Δ^ < 0.05 and *P*^ΔΔ^ < 0.01.

**Table 2:** Protein expression of FAK, PI3K, and Aggrecan of KOA rabbit articular cartilages ($\overset{-}{x}\pm s$).

Group	*N*	Protein expression of FAK	Protein expression of p-PI3K	Protein expression of Aggrecan
Control	6	0.06 ± 0.01	0.14 ± 0.02	1.54 ± 0.16
Model	6	0.45 ± 0.13^*∗∗*^	0.41 ± 0.18^*∗∗*^	0.59 ± 0.11^*∗∗*^
Model-inhibitor	6	0.15 ± 0.15^Δ^	0.22 ± 0.09^Δ^	0.19 ± 0.05^ΔΔ^
Needle knife	6	2.32 ± 0.32^*∗∗*##▲▲^	0.83 ± 0.19^*∗∗*##▲▲^	1.11 ± 0.16^*∗∗*##▲▲^
Needle knife-inhibitor	6	1.95 ± 0.35^Δ^	0.65 ± 0.18^Δ^	0.69 ± 0.08^ΔΔ^
Electroacupuncture	6	1.25 ± 0.28^*∗∗*##^	0.60 ± 0.17^*∗∗*#^	0.76 ± 0.14^*∗∗*#^
Electroacupuncture inhibitor	6	0.92 ± 0.23^Δ^	0.41 ± 0.15^Δ^	0.37 ± 0.13^ΔΔ^

Versus normal group: *P*^*∗∗*^ < 0.01; versus model group: *P*^#^ < 0.05 and *P*^##^ < 0.01; versus electroacupuncture group: *P*^▲▲^ < 0.01; versus noninhibitor group: *P*^Δ^ < 0.05 and *P*^ΔΔ^ < 0.01.
