# Supplementary material for: Effect of Acupotomy on FAK-PI3K Signaling Pathways in KOA Rabbit Articular Cartilages
Source: Evid Based Complement Alternat Med. 2017 Oct 12;2017:4535326. doi: 10.1155/2017/4535326 (PMC5660818; doi:10.1155/2017/4535326)
Supplement: Supplementary file 1 — Animal Experimental Ethical Inspection Form, Beijing University of Chinese Medicine. [file 4535326.f1.pdf]

Animal Experimental Ethical Inspection Form of Beijing University of Chinese Medicine

No.: Kj-dw-18-20150604-01

<table>
<tr><td rowspan="9">Concerned information wrote by applicant</td><td colspan="2">Applicant: Ma Shining</td><td>Educa0tion: Ph. D</td><td>Professional title:</td></tr>
<tr><td colspan="4">Experiment title: Effect of Acupotomy on FAK-PI3K signaling pathways in KOA rabbit articular cartilages</td></tr>
<tr><td colspan="4">Aim of experiment: The research of the effect of needle knife to promote cartilage cell synthesis metabolism mechanism</td></tr>
<tr><td rowspan="4">Animal cases</td><td colspan="3">Source of animal: Beijing Jin Muyang Laboratory Animal Technology Co. Ltd</td></tr>
<tr><td colspan="3">Species or strain: New Zealand rabbits Grade: SPF</td></tr>
<tr><td colspan="2">Number: 49</td><td>Application date: 2015-06-14</td></tr>
<tr><td colspan="2">Entering date: 2015-07-04</td><td>Ending date: 2015-09-24</td></tr>
<tr><td colspan="4">Outline of experiments: including modeling or surgical methods, observational index, executing animal method et al.
1. Modeling or surgical methods:
Modified Videman left hind leg unbend fixed braking method

2. Observational index:
RT-pcr and Western blot were used to test each animal cartilage p - FAK , p - PI3K, Aggrecan gene and protein expression level

3. Executing animal method:
Executed by over anesthesia.</td></tr>
<tr><td colspan="4">Signature of applicant: Ma Shining Telephone: 86 - 10 - 13426391690</td></tr>
<tr><td>Inspection contents</td><td colspan="4">1. Does laboratory animal must be used in the project? Could other methods such as computer simulation, cell culture or using the low-grade animal instead of the high-grade animal? Yes.

2. Are the qualification of applicant, species or strain, grade and specifications of animals suitable? Could the quantity of animals be reduced by improving the study design or using high quality animals? Yes

3. Could the study design and animal treatment be refined by ameliorating experimental method, adjusting observational index, executing animal method? Yes</td></tr>
<tr><td>Results of inspection</td><td colspan="4">Attitude of the Animal Care & Welfare Committee: | Agree ☐ | Disagree ☐ | Stamp: Zhaobaoshen ✓
Date:</td></tr>
</table>
